# Third dose of BNT162b2 improves immune response in liver transplant recipients to ancestral strain but not Omicron BA.1 and XBB

**DOI:** 10.3389/fimmu.2023.1206016

**Published:** 2023-07-03

**Authors:** Zi Wei Chang, Yun Shan Goh, Angeline Rouers, Siew-Wai Fong, Matthew Zirui Tay, Jean-Marc Chavatte, Pei Xiang Hor, Chiew Yee Loh, Yuling Huang, Yong Jie Tan, Vanessa Neo, Isaac Kai Jie Kam, Nicholas Kim-Wah Yeo, Eunice X. Tan, Daniel Huang, Bei Wang, Siti Nazihah Mohd Salleh, Eve Zi Xian Ngoh, Cheng-I. Wang, Yee-Sin Leo, Raymond Tzer Pin Lin, David Chien Boon Lye, Barnaby Edward Young, Mark Muthiah, Lisa F. P. Ng, Laurent Rénia, COVID-19 Study Group

**Affiliations:** ^1^ ASTAR Infectious Diseases Labs (ASTAR ID Labs), Agency for Science, Technology and Research (ASTAR), Singapore, Singapore; ^2^ National Public Health Laboratory, National Centre for Infectious Diseases, Singapore, Singapore; ^3^ Yong Loo Lin School of Medicine, National University of Singapore and National University Health System, Singapore, Singapore; ^4^ Division of Gastroenterology and Hepatology, Department of Medicine, National University Hospital, Singapore, Singapore; ^5^ National University Centre for Organ Transplantation, National University Health System, Singapore, Singapore; ^6^ Singapore Immunology Network (SIgN), Agency for Science, Technology and Research (ASTAR), Singapore; ^7^ Lee Kong Chian School of Medicine, Nanyang Technological University, Singapore, Singapore; ^8^ National Centre for Infectious Diseases (NCID), Singapore, Singapore; ^9^ Department of Infectious Diseases, Tan Tock Seng Hospital, Singapore, Singapore; ^10^ Saw Swee Hock School of Public Health, National University of Singapore, Singapore, Singapore; ^11^ Department of Microbiology and Immunology, Yong Loo Lin School of Medicine, National University of Singapore, Singapore, Singapore; ^12^ Department of Biochemistry, Yong Loo Lin School of Medicine, National University of Singapore, Singapore, Singapore; ^13^ Health Protection Research Unit in Emerging and Zoonotic Infections, National Institute of Health Research, University of Liverpool, Liverpool, United Kingdom; ^14^ Institute of Infection, Veterinary and Ecological Sciences, University of Liverpool, Liverpool, United Kingdom; ^15^ School of Biological Sciences, Nanyang Technological University, Singapore, Singapore

**Keywords:** SARS-CoV-2, spike protein, antibodies, T cells, immunosuppressives, BNT162b2, liver transplant recipients, B cells

## Abstract

Vaccine immunogenicity in transplant recipients can be impacted by the immunosuppressive (IS) regimens they receive. While BNT162b2 vaccination has been shown to induce an immune response in liver transplant recipients (LTRs), it remains unclear how different IS regimens may affect vaccine immunogenicity after a third BNT162b2 dose in LTRs, which is especially important given the emergence of the Omicron sublineages of SARS-CoV-2. A total of 95 LTRs receiving single and multiple IS regimens were recruited and offered three doses of BNT162b2 during the study period. Blood samples were collected on days 0, 90, and 180 after the first BNT162b2 dose. At each time point, levels of anti-spike antibodies, their neutralizing activity, and specific memory B and T cell responses were assessed. LTRs receiving single IS regimens showed an absence of poor immunogenicity, while LTRs receiving multiple IS regimens showed lower levels of spike-specific antibodies and immunological memory compared to vaccinated healthy controls after two doses of BNT162b2. With a third dose of BNT162b2, spike-specific humoral, memory B, and T cell responses in LTR significantly improved against the ancestral strain of SARS-CoV-2 and were comparable to those seen in healthy controls who received only two doses of BNT162b2. However, LTRs receiving multiple IS regimens still showed poor antibody responses against Omicron sublineages BA.1 and XBB. A third dose of BNT162b2 may be beneficial in boosting antibody, memory B, and T cell responses in LTRs receiving multiple IS regimens, especially against the ancestral Wuhan strain of SARS-CoV-2. However, due to the continued vulnerability of LTRs to presently circulating Omicron variants, antiviral treatments such as medications need to be considered to prevent severe COVID-19 in these individuals.

## Introduction

Since December 2019, coronavirus disease 2019 (COVID-19), caused by the severe acute respiratory syndrome coronavirus 2 (SARS-CoV-2), has had devastating effects on the global healthcare system and on society and the economy, with over 660 million clinical cases and ~6.6 million deaths reported worldwide (1). Among the different measures to mitigate the burden of COVID-19, mRNA-based vaccines have been the leading preventive interventions used to combat the disease. Initial trials conducted with healthy individuals demonstrated the induction of humoral and cellular responses by the mRNA-based vaccines BNT162b2 and mRNA-1273 (2, 3). However, solid organ transplant recipients, who are on immunosuppressive (IS) regimens to prevent transplant rejection, were not included in these trials. Nevertheless, vaccination of this population is recommended, and poor vaccine immunogenicity has been reported in solid organ transplant recipients, including liver transplant recipients (LTRs) (4, 5). Studies have shown reduced immunogenicity in LTRs compared to healthy controls (HC) after two doses of BNT162b2 (6–8), and IS regimens have been identified as risk factors for lower humoral and cellular responses in this population (9–11).

Although the risk of severe disease in breakthrough infection is lower in solid organ transplant recipients who have received two doses of BNT162b2 (12), a study in England showed higher risks of severe COVID-19 in solid organ transplant recipients during the Delta variant wave compared to the general population (13). Therefore, booster vaccination with a third dose of BNT162b2 is recommended for immunocompromised individuals, including transplant recipients. A recent study has demonstrated a significant improvement in humoral response in solid transplant recipients after three doses of BNT162b2 (14). IS regimens have been associated with differing BNT162b2 immunogenicity in several studies in LTRs (9, 10, 15). However, the direct impact of IS regimens on humoral and cellular responses in LTRs after three doses of BNT162b2 remains unknown. In this study, we aim to assess the impact of IS regimens on humoral and cellular responses of LTRs after three doses of BNT162b2 against the ancestral Wuhan strain and the Omicron sublineages BA.1 and XBB of SARS-CoV-2.

## Materials and methods

### Ethics statement and study population

The study design and protocol for the COVID-19 PROTECT study group were assessed by the National Healthcare Group (NHG) Domain Specific Review Board (DSRB) and approved under study number 2012/00917. Written informed consent was obtained from all study participants in accordance with the Declaration of Helsinki for Human Research. A cohort of 95 LTRs was recruited for the study. The interval between the first and second dose of BNT162b2 was 21 days (IQR: 21-24 days). On day 180, 61 out of the 95 LTRs had received a third dose of BNT162b2. The interval between the third dose of BNT162b2 and day 180 post first dose was 76 days (IQR: 54.5-97.75 days). The remaining 34 LTRs who did not receive the third dose and were COVID-19 positive before day 180 sampling were excluded. Blood collection was performed on days 0 (i.e., before the first BNT162b2 dose), 90 post first dose, and 180 post first dose. Blood samples from 268 age-matched healthy individuals who received two mRNA vaccines (BNT162b2) on day 90 post first dose were used as a control (HC). None of the individuals had known or reported SARS-CoV-2 infection and change in the IS regimens due to episodes of rejection or side effects of the medications.

### Commercial serological assays for the detection of anti-SARS-CoV-2 antibodies

The Elecsys^®^ Anti-SARS-CoV-2 S (Roche S) immunoassays were used to measure antibodies against the receptor-binding domain (RBD) of the spike protein. The Roche Cobas e411 Analyzer (Roche, Basel, Switzerland) was used for the assay according to the manufacturer’s instructions. The Roche S assay measured the electro-chemiluminescent signal representing antibody levels in titrated samples. Samples with antibody levels of ≥ 0.8 U/mL were considered positive.

### Spike protein flow cytometry-based assay (SFB assay) for antibody detection

The SFB assay was performed according to previously described methods (16, 17). In brief, cells expressing the spike protein of the ancestral Wuhan strain, Omicron BA.1 and XBB were seeded at 1.5 x 10^5^ cells/well in 96-well V-bottom plates (Thermo Fisher Scientific, Waltham, USA). The cells were incubated with human serum (diluted 1:100 in 10% FBS; HyClone, Chicago, USA), followed by a second incubation with a double stain comprising Alexa Fluor 647-conjugated anti-human IgG (diluted 1:500; Thermo Fisher Scientific) and propidium iodide (PI; diluted 1:2500; Sigma-Aldrich, Burlington, USA). Cells were acquired using an LSR4 laser (BD Biosciences, New Jersey, USA) and analyzed using FlowJo (Tree Star, BD Biosciences). The percentage of GFP-positive spike protein-expressing cells bound by the antibody, indicated by Alexa Fluor 647- and FITC-positive events, was used as an indicator of binding. The assay was performed as two independent experiments, each with technical duplicates. The amount of spike protein expressed on the cell surface was verified by ACE-2-HuFc binding. A subset of age-matched samples was randomly selected and examined for binding antibodies against Omicron BA.1 and XBB (n = 10 per LTRs with double and triple IS regimens and HC).

### Memory B cell ELISpot

SARS-CoV-2 RBD-specific memory B cell (MBC) numbers were quantified using the ELISpot Path: Human IgG (SARS-CoV-2, RBD) ALP kit (Mabtech, Cincinnati, USA) following the previously described protocol (18). PBMCs were suspended in RPMI + 10% FBS + 1 μg mL^-1^ R848 + 10 ng mL^-1^ IL-2 and incubated at 37°C for 5 days to allow for differentiation into antibody-secreting cells. To determine RBD-specific MBC numbers, 100,000 or 400,000 live cells were plated for ELISpot. Total IgG-secreting cells were detected by plating 1,500 or 3,000 live cells to normalize the results. Plates were then read on an IRIS ELISpot reader (Mabtech), and spots were quantified based on the average of duplicate wells. Due to limited cell availability, a subset of age-matched samples was randomly selected and examined (n = 42 for LTRs; n = 52 for HC).

### Extracellular and intracellular profiling of T cells with flow cytometry

SARS-CoV-2-specific T cell subsets were characterized using a previously described method with modifications (19, 20). PBMCs were rested overnight in RPMI-1640 + 5% human serum at 37 ˚C then stimulated with PMA (100ng/mL) (Sigma Aldrich) and ionomycin (1µg mL^-1^) (Sigma Aldrich) as a positive control or with pooled SARS-CoV-2 PepTivator^®^ S and S1 peptides (0.6nmol mL^-1^ each) (Miltenyi Biotec), or left unstimulated (baseline) for 6 h. Brefeldin A and Monesin (Thermo Fisher Scientific) were added at 2 h post stimulation. Cells were stained with surface markers for 30 min (**Table S1**, #1 to 21), fixed and permeabilized with Foxp3/Transcription Factor Staining Buffer Set (Thermo Fisher Scientific) for 30 min, and then stained for intracellular cytokines for another 30 min (**Table S2**, #22 to 29). Cells were acquired with the CytekTM Aurora (SpectroFlo^®^) and analyzed using FlowJo. Spike-specific intracellular granzyme B expression was determined after baseline subtraction. A subset of age-matched samples was randomly selected and examined due to limited cell availability (n = 41 for LTRs; n = 40 for HC).

### Pseudovirus neutralization assay

The pseudotyped lentivirus neutralization assay was performed according to a previously described protocol with slight modifications (21). Briefly, CHO-ACE2, a stable cell line expressing human ACE2, was acquired from Associate Professor Dr Yee-Joo Tan (Department of Microbiology, National University of Singapore & Institute of Molecular and Cell Biology, A*STAR, Singapore) (22) and utilized for the assay. The CHO-ACE2 cells were seeded at 1.8 x 10^4^ cells per well in a 96‐well black microplate (Corning, New York, USA) with DMEM without Geneticin and were allowed to settle overnight. Heat‐inactivated plasma samples were serially diluted (1:5 to 1:5120 dilutions) and incubated with an equal volume of pseudovirus-expressing spike proteins of the respective SARS-CoV-2 strain (5 ng of p24 per well) at 37°C for 1 h. The mixture was then added in duplicate to the pre‐seeded CHO‐ACE2 cells. The wells were topped up with DMEM after 1 h of incubation. After 48 h, cells were washed with phosphate-buffered saline (PBS) and lysed with 1X Passive Lysis Buffer (Promega) with gentle shaking at 125 rpm at 37°C for 30 min. Luciferase activity was subsequently quantified using the Luciferase Assay System (Promega) on a GloMax Luminometer (Promega). IC50 values were calculated as the reciprocal of the dilution at which a 50% reduction in luciferase activity was observed. A subset of age-matched samples was randomly selected and examined for binding antibodies against the spike protein of the ancestral Wuhan strain, as well as Omicron BA.1 and XBB (n = 10 per LTRs with double and triple IS regimens and HC).

### Statistical analysis

Statistical analysis was performed using GraphPad Prism 7. To compare different time points, unpaired comparisons were performed using the Mann-Whitney *U*-test. For the comparison between LTRs with different regimens and HC, the Kruskal-Wallis test was used, followed by *post hoc* tests. Dunn’s tests were used to correct for multiple comparisons. To compare between matched samples, the Wilcoxon matched-pairs signed-rank test was used. All tests were two-tailed, and *p* < 0.05 was considered statistically significant.

## Results

### Liver transplant recipients show enhancement of humoral and cellular responses following the administration of the third dose of the BNT162b2 vaccine

A cohort of 95 LTRs receiving various IS treatments (**Table S1**) were vaccinated with the Pfizer/BioNTech vaccine, BNT162b2, and their immune response was monitored. As LTRs are known to mount reduced antibody response (14, 23), the third dose of BNT162b2 was recommended for them. On day 180, 61 out of the 95 LTRs had received a third dose of BNT162b2, with a median interval of 76 days since the third dose (**Table S1**).

**Figure 1:**
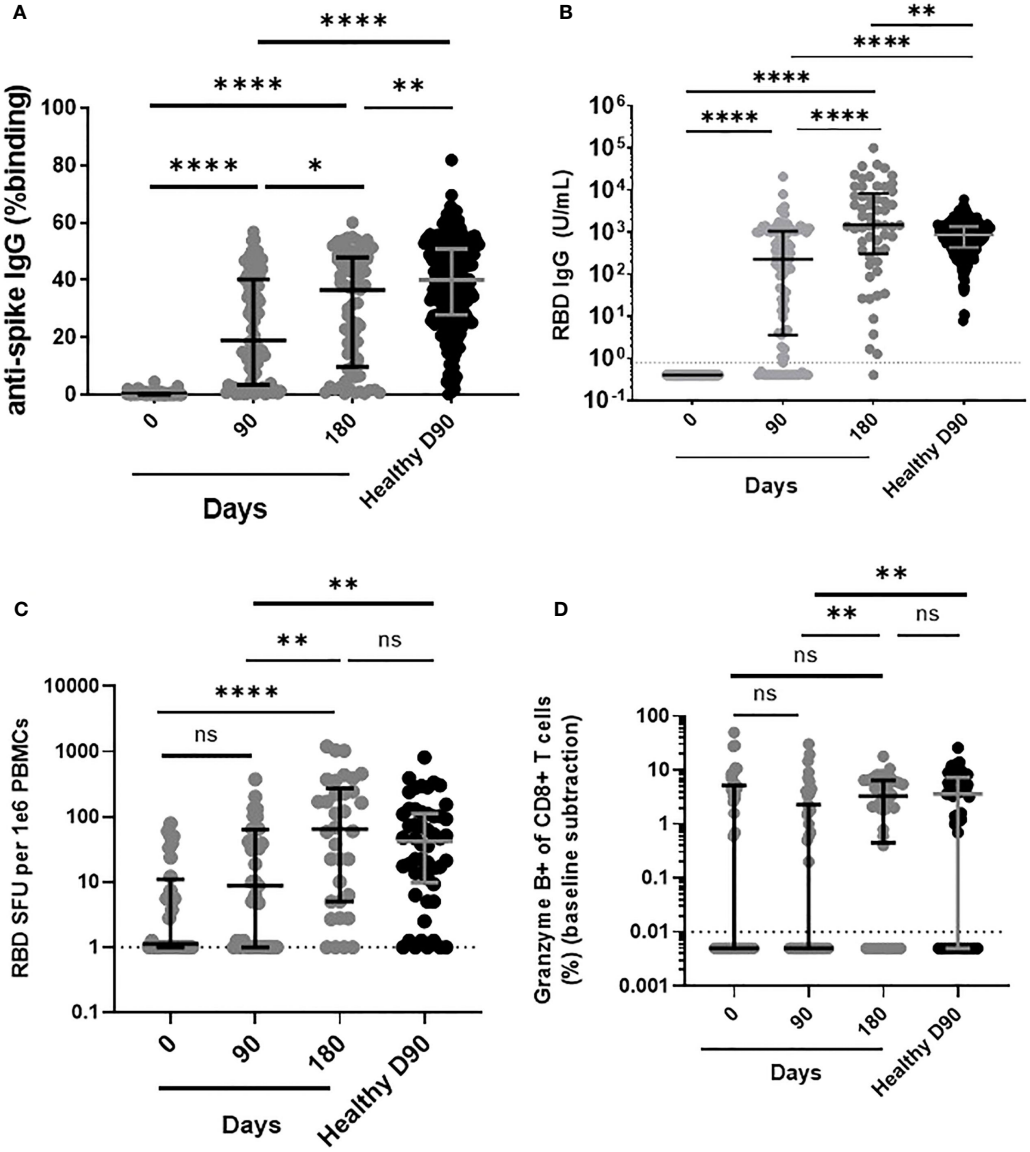
Comparison of humoral and cellular responses between LTRs vaccinated with two and three doses of BNT162b2 and HC vaccinated with two doses of BNT162b2. **(A)** Comparison of IgG responses against ancestral Wuhan strain full-length spike and **(B)** RBD in LTRs and HC (Median Age: LTR: 59 years, HC: 60 years) on days 0, 90, and 180 post first BNT162b2 dose. **(C)** Comparison of RBD-specific MBC among IgG+ Antibody Secreting Cells and **(D)** granzyme B-producing CD8 T cell responses in LTRs and HC on days 0, 90, and 180 post first dose of BNT162b2. *p < 0.05, **p < 0.01, ****p < 0.0001, (Mann-Whitney *U*-test). Data are presented as medians with interquartile range. ns, not significant.

After two doses of BNT162b2 (day 90), antibodies to full-length spike and RBD were induced and significantly increased in all LTRs, although the levels were significantly lower than those in HC on day 90 (**Figures 1A, B**). Following the third dose of BNT162b2, the level of antibodies against the full spike protein increased in LTRs but remained lower than in HC who received two doses (**Figure 1A**). In contrast, the level of antibodies against the RBD increased and was significantly higher than in HC who received two doses (**Figure 1B**).

Successful induction of humoral immune responses requires a well-coordinated response of B and T cells (24), with an effective CD8 cytotoxic T cell response being critical for eliminating the virus (25). To determine whether BNT162b2 induces spike-specific B and T cell recall responses, we next examined a subpopulation of vaccinated LTRs (n=42) due to limited cell availability. After two doses of BNT162b2, LTRs had significantly lower RBD-specific MBC response compared to HC vaccinated with two doses. However, after receiving the third dose of BNT162b2, the RBD-specific MBC response in LTRs increased and was similar to the response in HC vaccinated with two doses (**Figure 1C**).

To assess CD8+ T cell response, we measured the percentage of CD8+ T cells secreting granzyme B after SARS-CoV-2 peptide stimulation. After two doses of BNT162b2, the granzyme B-producing CD8 T cell response in LTRs remained significantly lower than in HC vaccinated on day 90. However, following the third dose of BNT162b2, a significant increase in the level of spike-specific granzyme B-producing CD8 T cells was observed in LTRs, becoming similar to the response of HC vaccinated with two doses (**Figure 1D**). Overall, LTRs required three doses of the vaccine to show robust antibody, MBC, and CD8+ T cell responses comparable to those seen in HC.

### Liver transplant recipients receiving two or more immunosuppressive drugs show diminished humoral, memory B, and T cell responses following two doses of the BNT162b2 vaccine

Calcineurin inhibitors are the most commonly prescribed IS drugs for maintenance immunosuppression in transplant recipients (26). However, there have been concerns regarding nephrotoxicity in transplant recipients when given at high doses (27). As a result, mycophenolate mofetil (MMF) and other IS drugs have been prescribed to replace or minimize the use of high calcineurin inhibitor doses in LTRs (28–30). Previous studies have demonstrated that an IS regimen with three different drugs or the use of MMF affects vaccine immunogenicity in LTRs (9, 11). Therefore, we stratified LTRs based on the number of received IS regimens and evaluated any differences in humoral and cellular responses after vaccination.

**Figure 2:**
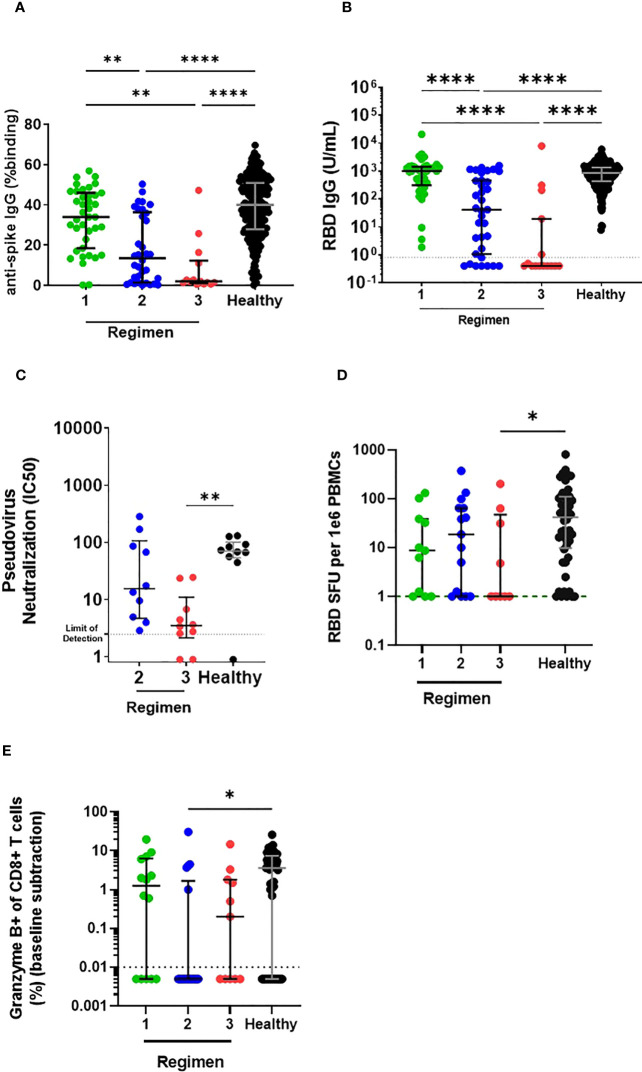
Comparison of humoral and cellular responses between LTRs receiving different immunosuppressive regimens and HC vaccinated with two doses of BNT162b2. **(A)** Comparison of IgG responses against ancestral Wuhan strain full-length spike and **(B)** RBD in stratified LTRs (LTRs receiving single, double, and triple IS regimens were labeled as regimens 1, 2, and 3, respectively.) and HC (Median Age: LTR Regimen 1: 59 years, LTR Regimen 2: 60 years, LTR Regimen 3: 57 years, HC: 61 years) on day 90 post first dose of BNT162b. **(C)** Comparison of neutralizing antibody response against ancestral Wuhan strain spike and in stratified LTRs and HC on day 90 post first dose of BNT162b2. **(D)** RBD-specific MBC among IgG+ Antibody Secreting Cells and **(E)** granzyme B-producing CD8 T cell responses in stratified LTRs and HC on day 90 post first dose of BNT162b2. *p < 0.05, **p < 0.01, ****p < 0.0001, (Kruskal-Wallis test). Data are presented as median with interquartile range.

We compared the different IS regimens after two vaccine doses and found that LTRs receiving a single IS regimen had significantly higher spike- and RBD-specific IgG antibodies than the stratified LTRs receiving multiple IS regimens. Spike- and RBD-specific IgG antibody levels of single IS LTRs did not differ from vaccinated HC. In contrast, LTRs receiving double and triple IS regimens had significantly lower spike- and RBD-specific IgG antibodies than vaccinated HC (**Figures 2A, B**). Moreover, LTRs receiving the triple IS regimen had significantly lower neutralizing antibodies against ancestral Wuhan spike compared to HC vaccinated with two doses (**Figure 2C**). Regarding MBC response, single and double IS regimens achieved an MBC response similar to vaccinated HC, while the triple IS regimen had a significantly lower MBC response (**Figure 2D**). For CD8+ T cell response, the percentage of granzyme B-producing CD8+ T cells was similar between the single IS regimen and HC. Only the double IS regimen had significantly lower levels of granzyme B-producing CD8 T cells compared to HC vaccinated with two doses (**Figure 2E**). Thus, LTRs receiving a single IS regimen did not show significantly different immune responses compared to HC, whereas LTRs receiving double and triple IS regimens had lower antibody, MBC, and CD8+ T cell responses.

### Liver transplant recipients receiving double and triple immunosuppressive regimens show enhancement of humoral, memory B, and T cell responses after the third dose of the BNT162b2 vaccine, similar to HC vaccinated with two doses

**Figure 3:**
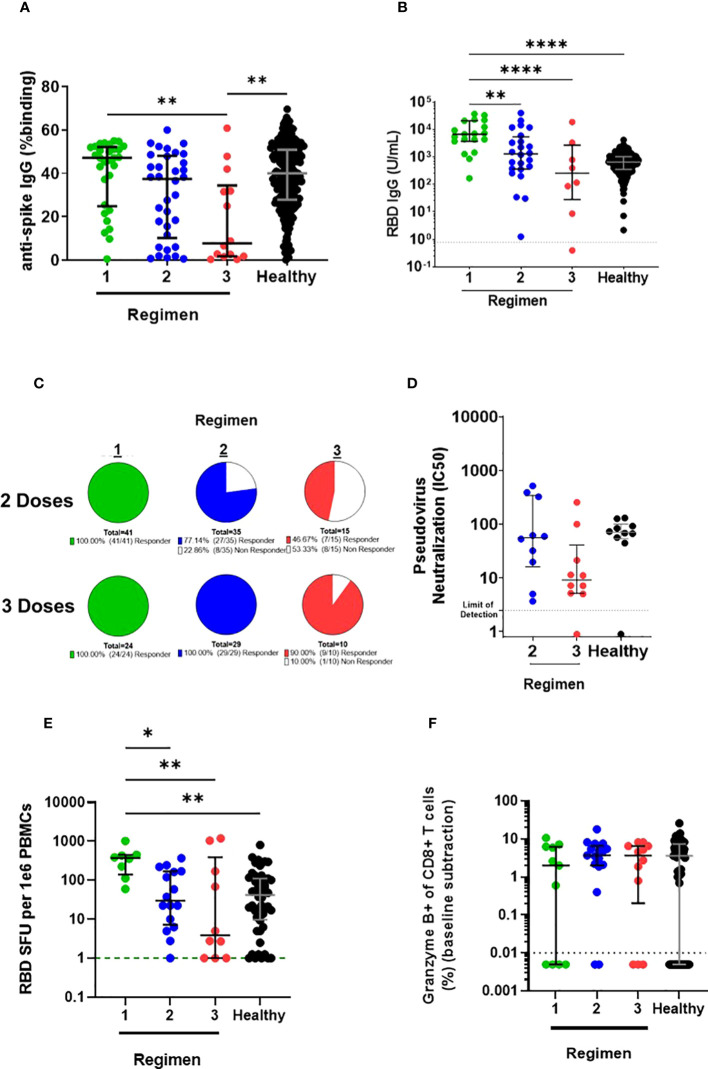
Comparison of humoral and cellular responses in LTRs receiving different immunosuppressive regimens vaccinated with a third dose of BNT162b2 and HC vaccinated with two doses of BNT162b2. **(A)** IgG responses against ancestral Wuhan strain full-length spike and **(B)** RBD in stratified LTRs on day 180 post first dose of BNT162b2 and HC on day 90 post first dose of BNT162b2. **(C)** The proportion of anti-RBD antibody responders and non-responders (<0.8U/mL RBD IgG) in stratified LTRs. **(D)** Neutralizing antibody response against ancestral Wuhan strain spike in stratified LTRs on day 180 post first dose of BNT162b2 and HC on day 90 post first dose of BNT162b2. **(E)** RBD-specific MBC among IgG+ Antibody Secreting Cells and **(F)** granzyme B-producing CD8 T cell responses in stratified LTRs on day 180 post first dose of BNT162b2 and HC on day 90 post first dose of BNT162b2. *p < 0.05, **p < 0.01, ****p < 0.0001, (Kruskal-Wallis test). Data are presented as median with interquartile range.

Next, we aimed to investigate whether a third dose of BNT162b2 could rescue the reduced immune responses observed in LTRs with double and triple IS regimens. After three doses of BNT162b2, LTRs with the double IS regimen had a similar level of antibodies against the full spike protein to HC with two vaccine doses, whereas LTRs with the triple IS regimen continued to have a significantly lower level of antibodies to the full spike protein compared to vaccinated HC (**Figure 3A**). Against the RBD protein, LTRs with double and triple IS regimens showed similar antibody levels to HC vaccinated with two doses (**Figure 3B**). Furthermore, the percentage of LTRs receiving multiple IS regimens who were antibody non-responders decreased 2-fold (from 22.86% to 0%) and 3-fold (from 53.3% to 10%) after three doses of BNT162b2 (**Figure 3C**). In addition, for LTRs receiving both double and triple IS regimens, neutralizing antibody responses were improved and were not significantly different compared to HC vaccinated with two doses of BNT162b2 (**Figure 3D**).

Similarly, after three doses of BNT162b2, both double and triple IS regimens showed similar MBC response to HC vaccinated with two doses, with no significant difference observed (**Figure 3E**). Additionally, the CD8+ T cell response in LTRs receiving double or triple IS regimens was boosted, with no significant difference in the percentage of granzyme B-producing CD8 T cells compared to HC vaccinated with two doses (**Figure 3F**). LTRs receiving a single IS regimen had significantly higher RBD-specific MBC response on day 180 than LTRs with multiple IS regimens or HC (**Figure 3E**). Although differences were observed in B cell response, all other LTR groups had comparable granzyme B-producing CD8 T cell responses after the third dose of BNT162b2 irrespective of the IS regimen (**Figure 3F**). Thus, a third dose of BNT162b2 rescued the reduced immune responses observed in LTRs with multiple IS regimens, leading to enhanced antibody, MBC, and CD8+ T cell responses that were similar to those observed in HC vaccinated with two doses.

### Liver transplant recipients on double and triple immunosuppressive regimens show diminished humoral response to Omicron BA.1 and XBB variants despite receiving the third dose of the BNT162b2 vaccine

Reduced antibody response has been observed against the emergent Omicron variant and its sublineages compared to the ancestral Wuhan strain in immunocompetent individuals (31, 32). Therefore, we aimed to investigate the cross-variant antibody breadth in LTRs receiving double and triple IS regimens after the third dose of the BNT162b2 vaccine against Omicron BA.1 and XBB variants.

**Figure 4:**
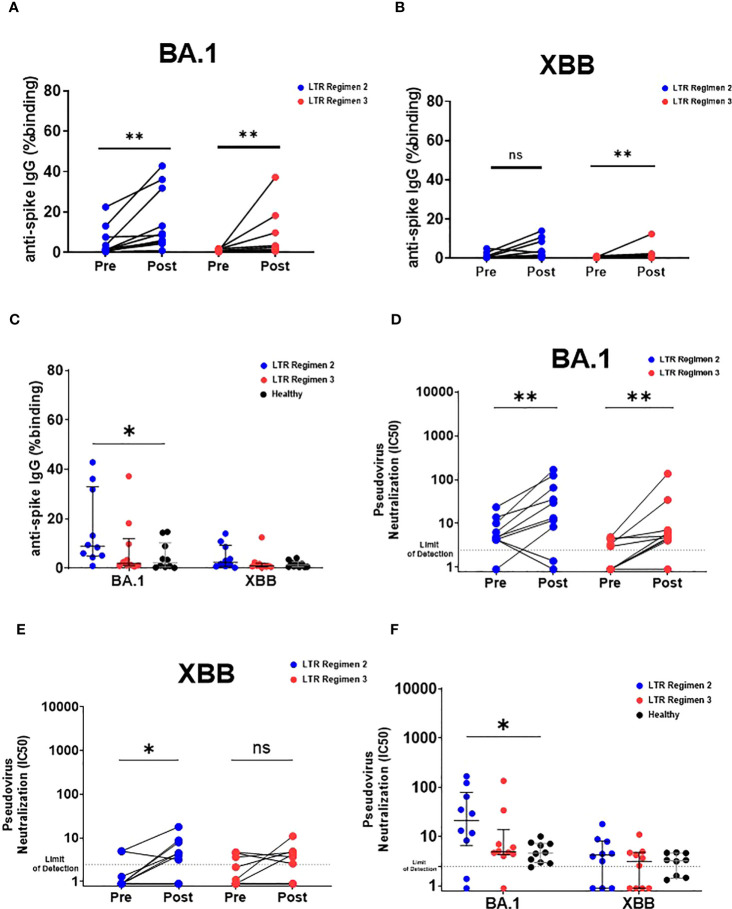
Humoral response against Omicron BA.1 and XBB in LTRs after receiving the third dose of BNT162b2. IgG responses against **(A)** Omicron BA.1 and **(B)** Omicron XBB spike in stratified LTRs receiving double and triple IS regimens before and after the third dose of BNT162b2. **p < 0.01, (Wilcoxon matched-pairs signed rank test). **(C)** Comparison of IgG responses against Omicron BA.1 and Omicron XBB spike between stratified LTRs receiving double and triple IS regimens on day 180 post first dose of BNT162b2 and HC on day 90 post first dose of BNT162b2. Neutralizing antibody response against **(D)** Omicron BA.1 and **(E)** Omicron XBB spike in stratified LTRs receiving double and triple IS regimens before and after the third dose of BNT162b2. *p < 0.05, **p < 0.01, (Wilcoxon matched-pairs signed-rank test). **(F)** Neutralizing antibody response against Omicron BA.1 and Omicron XBB spike in stratified LTRs receiving double and triple IS regimens on day 180 post first dose of BNT162b2 and HC on day 90 post first dose of BNT162b2. *p < 0.05, **p < 0.01, (Kruskal-Wallis test). Data are presented as median with interquartile range. ns, not significant.

Binding antibody responses against Omicron sublineages increased after the third dose of BNT162b2 for LTRs receiving both double and triple IS regimens (**Figures 4A, B**). Interestingly, LTRs with the double IS regimen showed higher antibody response against Omicron BA.1 than HC vaccinated with two doses (**Figure 4C**). Similarly, neutralizing antibodies were boosted against Omicron BA.1 (**Figure 4D**), although neutralization activity against XBB remained very low (**Figure 4E**). Notably, LTRs with double but not triple IS regimens showed higher antibody response against Omicron BA.1 than HC vaccinated with two doses (**Figure 4F**). Thus, the three-dose regimen in LTRs provided a cross-variant antibody breadth similar to that of HC vaccinated with two doses of BNT162b2.

## Discussion

In a cohort of LTRs, we confirmed that multiple IS regimens were associated with suboptimal antibody, MBC, and CD8+ T cell responses after two doses of BNT162b2 compared to individuals receiving one IS regimen. Our findings are consistent with other studies that have also reported lower humoral and cellular responses in LTRs receiving multiple IS regimens (9, 33, 34). A third dose of BNT162b2 rescued the suboptimal immune responses observed in LTRs with double and triple IS regimens, leading to enhanced antibody, MBC, and CD8+ T cell responses that were similar to those observed in HC vaccinated with two doses. Liver transplant recipients on multiple immunosuppressive regimens show diminished humoral response to Omicron BA.1 and XBB variants despite receiving the third dose of the BNT162b2 vaccine.

Our findings agree with recent studies, which reported an increase in antibody and T cell response in LTRs after receiving a third dose of BNT162b2 (23, 35–37). Unlike these studies, our study demonstrates that T cell responses in LTRs receiving multiple IS regimens were similar to single IS despite the difference in humoral and MBC response after a third dose of BNT162b2. IFNγ CD8 T cell response was also assessed, but no difference was observed among the different regimens. Interestingly, individuals receiving a single IS regimen did not show poor immunogenicity after two doses of BNT162b2. Therefore, our study sheds light on the implementation of single IS regimen prior to vaccination in LTRs to maximize vaccine efficacy. However, individuals receiving a single IS regimen had a uniform CD8+ T cell response but no humoral or MBC response after the third vaccine dose, unlike those receiving multiple IS regimens. It is possible that this observation is due to the plateau in the CD8+ T cell response after the third dose of BNT162b2, as observed in (35). Hence, our study provides insight into the presence of T cell response in those receiving multiple IS regimens and warrants further investigation to determine the impact of single versus multiple IS treatments in T cell responses after additional boosters.

In agreement with several studies in solid organ transplant recipients (38, 39), our study demonstrates an increase in humoral response to Omicron BA.1 after the third dose of BNT162b2. The increase in the cross-variant antibody breadth can be extended to the Omicron XBB, as demonstrated in our study. Studies have shown that antibodies and T cell response are associated with risk against breakthrough infection and severe disease in LTRs and kidney transplant recipients (40–42). However, the diminished humoral responses against Omicron and its sublineages observed in individuals receiving multiple IS regimens can confer sufficient protection against the presently circulating Omicron variants and future SARS-CoV-2 variants remained to be determined.

Our study has a few limitations. Firstly, the comparison group of vaccinated HC only received two doses of BNT162b2, as the recommendation for a third dose of BNT162b2 was limited to immunocompromised individuals at the time of the study. Although we did not observe any differences in the B and T cell responses between LTRs vaccinated with three doses and HC vaccinated with two doses, it remains to be determined whether LTRs who are given a fourth dose would show the same quality of immune response as HC receiving a third dose of BNT162b2. Secondly, it remains uncertain whether the increased immune responses after the third vaccine dose were long-lasting and provided protection against infection and severe COVID-19 beyond the last time point in our study, which was a median of 76 days (IQR: 54.5-97.75days) post third dose (**Table S1**). Notably, kidney transplant recipients have shown waning antibody and T cell responses 6 months after receiving the third dose of BNT162b2 (43), and it remains to be seen whether LTRs show a similar trend. Shorter intervals between transplantation and the first dose of BNT162b2 have been associated with poor immunogenicity (15). Although all LTRs recruited had no episodes of rejection, single IS LTRs had a longer interval between their transplant and the first dose of BNT162b2 (**Table S1**), and this could have also contributed to the enhanced immunogenicity in comparison to the multiple IS LTRs. Additionally, there were more female LTRs receiving triple IS than other IS regimens (**Table S1**). A total of 5 out of 15 triple IS LTRs had autoimmune hepatitis and most of these patients with autoimmune hepatitis were female and required higher doses of immunosuppression post transplant (44). Further studies are required to address whether sex can influence immunogenicity in multiple IS LTRs.

Our study sheds light on the impact of multiple IS regimens on the humoral and cellular response in BNT162b2-vaccinated LTRs and highlights the increased vulnerability of this patient population to COVID-19. Our findings further emphasize the efficacy of additional vaccine doses in LTRs receiving multiple IS regimens and can guide better management of COVID-19 in this population. In addition to vaccination, antiviral treatments such as medications may be needed to ensure that vulnerable LTRs with poor immune responses remain protected from severe COVID-19.

## Data availability statement

The original contributions presented in the study are included in the article/**Supplementary Material**. Further inquiries can be directed to the corresponding author.

## Ethics statement

The studies involving human participants were reviewed and approved by National Healthcare Group, Singapore. The patients/participants provided their written informed consent to participate in this study.

## Author contributions

ZC, AR, YG, S-WF, and MT: conceptualized the study, designed and performed the experiments, analyzed the data, and wrote the manuscript. J-MC: designed and performed the experiments and analyzed the data. YT, PH, CL, YH, IK, NY, VN, YH, and SS: performed the experiments and analyzed the data. BW, SS, EN, and C-IW: designed and validated the pseudovirus neutralization assay and provided resources for the assay. ET, DH and MM: supervised and coordinated cohort recruitment and sample collection. COVID-19 Cohort Study Group: processed samples. Y-SL, RL, DL, BY, LN, and LR: conceptualized the study and reviewed the manuscript. All authors approved the final version of the manuscript. The team authors of the COVID-19 study group are listed in **Table S3**.
