# Supplementary material for: Third dose of BNT162b2 improves immune response in liver transplant recipients to ancestral strain but not Omicron BA.1 and XBB
Source: Front Immunol. 2023 Jul 3;14:1206016. doi: 10.3389/fimmu.2023.1206016 (PMC10350672; doi:10.3389/fimmu.2023.1206016)
Supplement: Supplementary file 1 [file Table_1.docx]

**Table S1. Demographics of liver transplant recipients.**

|  | Liver Transplant Recipients (LTRs) |  |  |  | Healthy Controls (HC) |
| --- | --- | --- | --- | --- | --- |
|  |  | Regimen | | |  |
|  |  | 1 | 2 | 3 |  |
| **N** | 95 | 42 | 38 | 15 | 268 |
|  |  |  |  |  |  |
| **Demographics** |  |  |  |  |  |
| Age, median (IQR), years | 59 (46-66) | 59 (43-66) | 60 (49-65.75) | 57 (45-62.5) | 60 (34-68) |
| Male sex, n (%) | 62 (65%) | 29 (69%) | 28 (74%) | 5 (33%) | 114 (43%) |
| Interval between 1^st^ transplantation and 1^st^ dose of BNT162b2, median (IQR), years | 6.0 (3.2-11.1) | 9.6 (6.7-17.2) | 3.6 (2.4-5.7) | 3.0 (1.1-7.4) | N.A. |
|  |  |  |  |  |  |
| **Vaccination** |  |  |  |  |  |
| Received 3^rd^ dose of BNT162b2 | 61 | 21 | 29 | 11 | N.A. |
| Interval between blood sampling at Day 180 and 1^st^ dose of BNT162b2, median (IQR) | 179 (166-207) | 202 (165-242) | 179 (169-196) | 166 (163.5-181) | N.A. |
| Interval between 3^rd^ dose of BNT162b2 and day 180, median (IQR) | 76 (54.5-97.75) | 90 (75-104) | 72 (56-97.5) | 58 (47-69.5) | N.A. |
|  |  |  |  |  |  |
| **Immunosuppressive (IS)** |  |  |  |  |  |
| Calcineurin inhibitor | 90 (92%) | 38 (90%) | 37 (97%) | 15 (100%) | N.A. |
| Tacrolimus Trough Levels at 1^st^ dose of BNT162b2, median (IQR) | 3.85 (3.1-5.775) | 3.2 (2.6-3.5) | 4.9 (3.45-5.7) | 6 (4.5-6.95) | N.A. |
| Steroid | 16 (16%) | 0 (0%) | 2 (5%) | 14 (93%) | N.A. |
| MMF^a^ | 40 (41%) | 1 (2%) | 27 (71%) | 12 (80%) | N.A. |
| Other (e.g., IL2 receptor mAb, mTOR inhibitor, etc.) | 16 (16%) | 3 (7%) | 9 (23%) | 4 (27%) | N.A. |
|  |  |  |  |  |  |
| ‡Double immunosuppression (Regimen 2) |  |  |  |  |  |
| Calcineurin inhibitor + MMF |  |  | 27 (71%) |  |  |
| Calcineurin inhibitor + Steroid |  |  | 2 (5%) |  |  |
| Calcineurin inhibitor + Other |  |  | 8 (21%) |  |  |
| Other |  |  | 1 (2%) |  |  |
|  |  |  |  |  |  |
| ‡‡Triple immunosuppression (Regimen 3) |  |  |  |  |  |
| Calcineurin inhibitor + MMF + Steroid |  |  |  | 11 (73%) |  |
| Calcineurin inhibitor + MMF + Others |  |  |  | 1 (7%) |  |
| Calcineurin inhibitor + Steroid +Other |  |  |  | 3 (20%) |  |
| ^a^ Mycophenolate Mofetil | | | | | |

**Table S2. Flow cytometry antibodies for SARS-CoV-2-specific T cells by Intracellular Cytokine Staining (ICS).**

| **No.** | **Marker** | **Colour** | **Volume (**μ**l)** | **Clone** | **Cat. No.** | **Vendor** |
| --- | --- | --- | --- | --- | --- | --- |
| 1 | CD66B | BV421 | 2 | G10F5  | 562940 | BD Biosciences |
| 2 | CD45RA | SB436 | 2 | H100 | 62-0458-42 | Thermo Fisher |
| 3 | CD27 | PB | 1 | O323 | 302822 | Biolegend |
| 4 | NKG2C | BV480 | 2 | 134591 | 748168 | BD Biosciences |
| 5 | CD8 | BV605 | 1 | SK1 | 564116 | BD Biosciences |
| 6 | CD19 | BV605 | 1 | HIB19 | 740394 | BD Biosciences |
| 7 | VD2 | BV711 | 1 | B6 | 331412 | Biolegend |
| 8 | CD107A | BV786 | 2 | H4A3 | 563869 | BD Biosciences |
| 9 | NKP46 | BB515 | 2 | 9-e2 | 564536 | BD Biosciences |
| 10 | CD3 | SB550 | 1 | SK7 | 344852 | Biolegend |
| 11 | CD169 | PERCP5.5 | 5 | 7-239 | 346020 | Biolegend |
| 12 | HLADR | AF700 | 2 | L243 | 307626 | Biolegend |
| 13 | CD4 | SN685 | 1 | SK3 | 344658 | Biolegend |
| 14 | CD14 | APC CY7 | 1 | M0P9 | 557831 | BD Biosciences |
| 15 | VD1 | APC Vio770 | 1 | REA173 | 130-120-578 | Miltenyi Biotec |
| 16 | L/D | ZOMBIE NIR | 0.5 |  | 423105 | Biolegend |
| 17 | CXCR5 | PE/Dazzle™ 594 | 1 | J252D4 | 356928 | Biolegend |
| 18 | CD154 | PE CY5 | 5 | TRAP-1 | 555701 | BD Biosciences |
| 19 | CD56 | PE CY5.5 | 2 | NCAM16.2 | 35-0567-42 | Thermo Fisher |
| 20 | CD16 | PE/Fire™ 640 | 0.5 | 3G8 | 302068 | Biolegend |
| 21 | CD45 | BUV805 | 2 | H130 | 612891 | BD Biosciences |
| 22 | Granzyme B | BV510 | 2.5 | GB11 | 563388 | BD Biosciences |
| 23 | IFN-y | BV570 | 2.5 | 4S.B3 | 502534 | Biolegend |
| 24 | IL-2 | BV650 | 5 | MQ1-17H12 | 564166 | BD Biosciences |
| 25 | TNF-a | BV750 | 2.5 | MAB11 | 566359 | BD Biosciences |
| 26 | IL-6 | FITC | 2.5 | MQ2-13A5 | 11-7069-82 | Thermo Fisher |
| 27 | IL-17a | AF647 | 10 | N49-653 | 560490 | BD Biosciences |
| 28 | IL-10 | PE | 2.5 | JES-9D7 | 501404 | Biolegend |
| 29 | IL-4 | PECY7 | 2.5 | 8D4-8 | 560672 | BD Biosciences |

**Table S3: COVID-19 Study Group team authors.**

| **Name** | **Highest Degree** | **Affiliation / Institution** |
| --- | --- | --- |
| Adeline C.Y. Chua | PhD | A*STAR Infectious Diseases Labs, Agency for Science, Technology and Research |
| Samantha Y. T. Nguee | PhD | A*STAR Infectious Diseases Labs, Agency for Science, Technology and Research |
| Guillaume Carissimo | PhD | A*STAR Infectious Diseases Labs, Agency for Science, Technology and Research |
| Anthony Torres-Ruesta | PhD | A*STAR Infectious Diseases Labs, Agency for Science, Technology and Research |
| Nathan Wong | PhD | A*STAR Infectious Diseases Labs, Agency for Science, Technology and Research |
| Siti Naqiah Amrun | BSc | A*STAR Infectious Diseases Labs, Agency for Science, Technology and Research |
| Wendy Yehui Chen | MSc | A*STAR Infectious Diseases Labs, Agency for Science, Technology and Research |
| Alice Soh Meoy Ong | BSc | A*STAR Infectious Diseases Labs, Agency for Science, Technology and Research |
| Estelle Yi Wei Goh | BSc | A*STAR Infectious Diseases Labs, Agency for Science, Technology and Research |
